# *O*-GlcNAc transferase regulates p21 protein levels and cell proliferation through the FoxM1–Skp2 axis in a p53-independent manner

**DOI:** 10.1016/j.jbc.2022.102289

**Published:** 2022-07-20

**Authors:** Rafaela Muniz de Queiroz, Sung-Hwan Moon, Carol Prives

**Affiliations:** Department of Biological Sciences, Columbia University, New York, New York, USA

**Keywords:** cell cycle, *O*-GlcNAcylation, OGT, protein degradation, p53, p21, CDK, cyclin dependent kinase, OGA, *O*-GlcNAcase, OGT, *O*-GlcNAc transferase, PI, propidium iodide, SCF, Skp-Cullin-F-box, TMG, Thiamet-G

## Abstract

The protein product of the CDKN1A gene, p21, has been extensively characterized as a negative regulator of the cell cycle. Nevertheless, it is clear that p21 has manifold complex and context-dependent roles that can be either tumor suppressive or oncogenic. Most well studied as a transcriptional target of the p53 tumor suppressor protein, there are other means by which p21 levels can be regulated. In this study, we show that pharmacological inhibition or siRNA-mediated reduction of *O*-GlcNAc transferase (OGT), the enzyme responsible for glycosylation of intracellular proteins, increases expression of p21 in both p53-dependent and p53-independent manners in nontransformed and cancer cells. In cells harboring WT p53, we demonstrate that inhibition of OGT leads to p53-mediated transactivation of CDKN1A, while in cells that do not express p53, inhibiting OGT leads to increased p21 protein stabilization. p21 is normally degraded by the ubiquitin-proteasome system following ubiquitination by, among others, the E3 ligase Skp-Cullin-F-box complex; however, in this case, we show that blocking OGT causes impairment of the Skp-Cullin-F-box ubiquitin complex as a result of disruption of the FoxM1 transcription factor–mediated induction of Skp2 expression. In either setting, we conclude that p21 levels induced by OGT inhibition correlate with cell cycle arrest and decreased cancer cell proliferation.

Cell cycle arrest is triggered in response to stabilization of p53 and transcriptional activation of its target gene *CDKN1A*, which encodes the cyclin-dependent kinase (CDK) inhibitor p21 (1). p21 (as well as the related CDK inhibitor, p27) prevents the G1/S transition by inhibiting the CDK2–cyclin E complex, while the G2/M transition can be regulated by p21 and p27 inhibition of the CDK1–cyclin A complex (2). While p53 is the most well validated regulator of p21 gene expression (3, 4, 5, 6), levels of p21 protein can be induced by p53-independent pathways (4, 7, 8, 9, 10). For example, the half-life of p21 is regulated by different protein complexes depending on p21 subcellular localization and its posttranslational modification status (11). One of those complexes is the Skp-Cullin-F-box (SCF)–ubiquitin complex ligase complex (12, 13), which, when associated with its cofactor Skp2 targets p21 as well as p27 for degradation; and the SCF-ubiquitin complex has been associated with modulation of the cell cycle (6, 14, 15, 16).

A common carbohydrate modification of intracellular proteins, *O*-GlcNAcylation, is the addition of a single GlcNAc moiety to serine and threonine residues in proteins. Similar to what is known about protein phosphorylation, *O*-GlcNAcylation can regulate protein interactions, subcellular localization, and enzymatic activity (17, 18). *O*-GlcNAc transferase (OGT) is the sole enzyme that is known to catalyze the addition of GlcNAc to intracellular proteins in cells. The removal of GlcNAc is performed by a second enzyme, *O*-GlcNAcase (OGA). OGT is needed for cell viability in mammals, and deletion of OGT leads to embryo lethality in flies, zebrafish, and mice (19, 20). Glycosylation, including *O*-GlcNAcylation, is aberrant in cancer cells and this can contribute in different ways to tumorigenesis (17, 21).

Modulation of both OGT and OGA can stabilize p53 and activate the p53 pathway in tumor cells (22, 23). Among the effects of *O*-GlcNAc modulation are increased levels of p21. Since p53 is not the only mode by which p21 is regulated in cells, we sought to elucidate the mechanism(s) involved in the modulation of p21 by *O*-GlcNAc and to determine whether p53 is the only route by which this enzyme may regulate p21.

## Results

### Inhibition of OGT but not OGA induces p21 expression independently of p53 status and cell type

**Figure 1:**
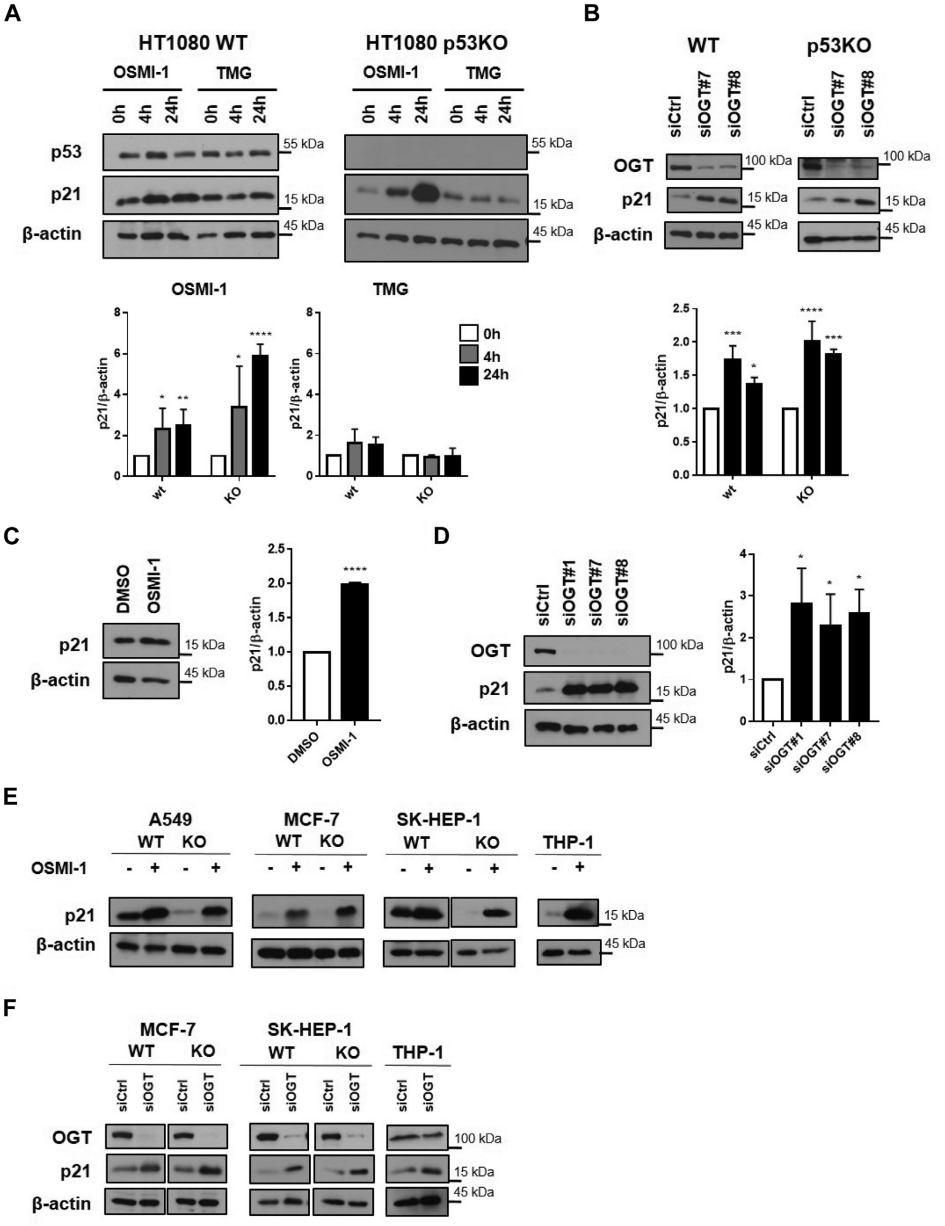
**Inhibition of OGT but not of OGA regulates p21 levels independently of p53.***A* and *B*, protein levels of p21 in HT1080 parental cells harboring WT p53 or p53-KO cells in response to (*A*) treatment with OGT inhibitor (OSMI-1, 50 μM) or OGA inhibitor (TMG; 10 μM) or (*B*) in response to silencing of OGT with siRNAs. Note that in (*A*), extracts of TMG-treated p53KO cells that were processed for immunoblotting are from the same gel as that shown in Fig. S1*B*. Note that in (*B*), extracts of p53KO cells that were processed for immunoblotting are from the same gel as that shown in Fig. S1*C*. *C* and *D*, protein levels of p21 in the p53 null cell line H1299 in response to (*C*) treatment with OSMI-1 (50 μM, 24 h) or (*D*) silencing of OGT with siRNAs. *E*, protein levels of p21 in the indicated cell lines harboring WT p53 or p53-KO derived cells in response to treatment with OSMI-1 (50 μM, 24 h). There were no THP-1 p53KO cells available for these experiments. *F*, p21 protein levels in cells harboring WT p53 or p53-KO cells as in (*E*) where OGT was silenced using siRNA. Bar graphs in (*A*–*D*) were derived by combining biological replicates where p21 protein levels were normalized to β-actin. Experiments shown represent at least three biological replicates, ∗*p* < 0.05, ∗∗*p* < 0.01, ∗∗∗*p* < 0.001, ∗∗∗∗*p* < 0.0001. OGA, *O*-GlcNAcase; OGT, *O*-GlcNAc transferase.

Modulation of *O*-GlcNAcylation leads to activation of p53 and expression of p21 in cancer cells (22, 24). Since p21 can also be induced by mechanisms independently of p53, however, we determined whether p53 is needed for the effects of *O*-GlcNAcylation on p21 levels. To address this, we used the OGT inhibitor, OSMI-1, and the OGA inhibitor, Thiamet-G (TMG), in HT1080 fibrosarcoma cells harboring WT p53 (HT1080 WT) and compared their response to CRISPR-engineered HT1080 cells in which p53 expression was absent (HT1080 p53KO). While OGA inhibition led to accumulation of *O*-GlcNAcylated proteins (Fig. S1*B*), it did not produce any changes in p21 levels in HT1080 WT cells (Fig. 1*A*). Treatment with the OGT inhibitor on the other hand, which decreased levels of GlcNAc-modified proteins (Fig. S1*A*), led to increased expression of p21 (Fig. 1*A*), an expected result based on previous evidence (22). Surprisingly, when HT1080 p53KO cells were treated with OSMI-1, there was a more dramatic induction of p21 protein, while OGA inhibition did not change p21 expression in these cells (Fig. 1*A*).

To confirm specificity and extend the effects of OSMI-1 on p21 expression, we targeted OGT using siRNA (Fig. S1*C*). As observed in OSMI-1–treated cells, silencing of OGT led to increasing levels of p21 in both WT and p53KO cell lines (Fig. 1*B*). Further, a different OGT inhibitor produced the same effect as OSMI-1 in both WT and p53KO cell lines (Fig. S1*D*).

We then moved to the H1299 lung carcinoma cell line that intrinsically lacks p53 expression. Here too, OGT inhibition, either using the pharmacological inhibitor, OSMI-1, or siRNA against OGT led to increased p21 levels in H1299 cells (Fig. 1, *C* and *D*).

To extend our findings to other cell types, we used a breast cancer cell line (MCF-7), an endothelial-derived liver cancer cell line (SK-HEP-1), a lung adenocarcinoma cell line (A549), and a monocytic-like cell line THP-1 (noncancer cells). These cell lines harbor WT p53. Further, with the exception of THP-1 cells, we had also generated CRISPR p53KO cell lines from these cell lines (25). OGT inhibition by OSMI-1 (Fig. 1*E*) or siRNA (Fig. 1*F*) led to increased levels of p21 in all cell lines tested, including the aforesaid CRISPR p53KO-derived cells. Additionally, we treated cells harboring different p53 mutations with OSMI-1, and OGT inhibition still induced expression of p21 independent of the p53 mutant present in cells (Fig. S1*E*). We conclude that the effect of OGT modulation in p21 expression is not cell-type specific and that p53 is not needed for this effect in any cell type that we tested.

### OGT decreases cell proliferation and induces cell cycle arrest through p21 independently of p53

We aimed to investigate if the effects of OGT inhibition on p21 levels have a functional relevance for cell dynamics. Although the presence of p53 did not have a significant impact on the effects of OGT inhibition on p21 levels, p53 affects cell proliferation, cell death, and the cell cycle. Thus, to eliminate this complexity, we used HT1080 p53KO cells for the initial analysis of OGT inhibition on cell viability and proliferation.

**Figure 2:**
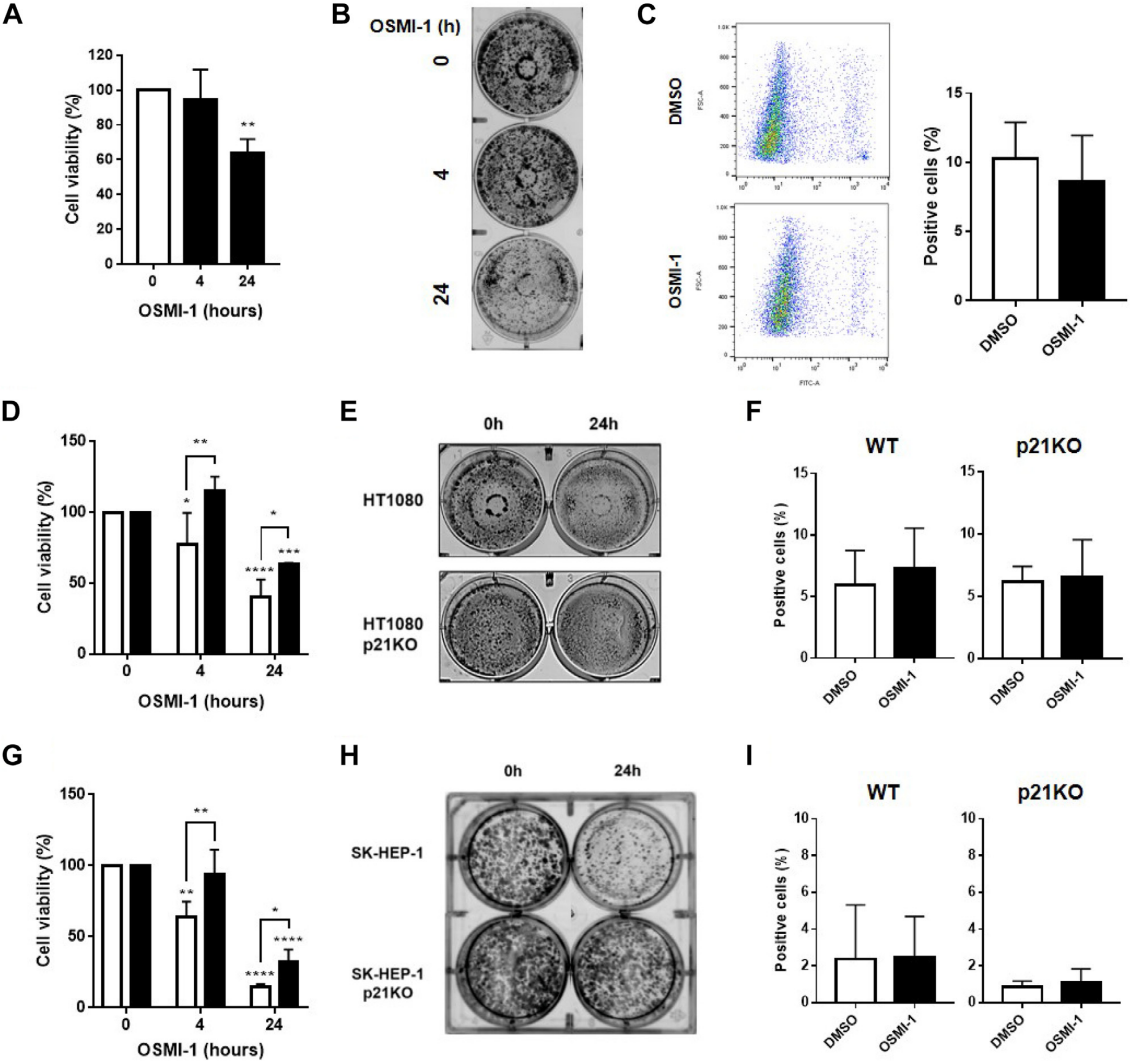
**OGT inhibition decreases cell proliferation without inducing cell death through modulation of p21.***A*, cell viability in response to OSMI-1 (50 μM) treatment in HT1080 p53KO cells for the indicated time periods measured by CellTiter-Glo. *B*, colony formation in response to OSMI-1 treatment for the indicated time periods in HT1080 p53KO cells. *C*, quantification of dead cells (SYTOX green positive) in response to treatment with OSMI-1 for 24 h in HT1080 p53KO cells. Dot plots represent positive and negative cells for dead/alive dye for each condition. *D*, cell viability in response to OSMI-1 treatment as in (*A*) in HT1080 cells (*white bars*) and HT1080 p21KO cells (*black bars*). *E*, colony formation in response to OSMI-1 treatment as in (*B*) in HT1080 cells and HT1080 p21KO cells. *F*, quantification of dead cells (SYTOX green positive) in response to treatment with OSMI-1 for 24 h in HT1080 cells and HT1080 p21KO cells as in (*C*). *G*, cell viability in response to OSMI-1 treatment as in (*A*) in SK-HEP-1 cells (*white bars*) and SK-HEP-1 p21KO cells (*black bars*). *H*, colony formation in response to OSMI-1 treatment as in (*B*) in SK-HEP-1 cells and SK-HEP-1 p21KO cells. *I*, quantification of dead cells (SYTOX green positive) in response to treatment with OSMI-1 for 24 h in SK-HEP-1 cells and SK-HEP-1 p21KO cells as in (*C*). Quantification plots correspond to different biological replicates combined. Experiments shown represent at least three biological replicates, ∗*p* < 0.05, ∗∗*p* < 0.01, ∗∗∗*p* < 0.001, ∗∗∗∗*p* < 0.0001. OGT, *O*-GlcNAc transferase.

When HT1080 p53KO cells were treated with OSMI-1, viability was significantly reduced as measured by cellular ATP levels (Fig. 2*A*). The decrease in cell viability could be due to either reduced cell proliferation or increased cell death. After performing a colony formation assay (Fig. 2*B*) and cell death assay (Fig. 2*C*), we observed that there was decreased cell proliferation with no significant induction of cell death in response to OSMI-1 treatment. The same reduction in cell proliferation with no change in cell death induction was observed in H1299 cells (Fig. S3, *A* and *C*). Treatment with the OGA inhibitor, TMG, did not induce significant changes in cell proliferation or cell viability (Fig. S2, *A* and *B*).

To determine whether p21 modulation is responsible for the observed changes in cell viability, we turned to use HT1080 cells that were engineered *via* CRISPR-Cas9 to lack expression of p21 (HT1080 p21KO) (Fig. S2*C*). The decrease in cell viability observed after treatment with OSMI-1 in HT1080 cells was completely rescued at the 4 h time point and partially rescued at the 24 h time point in the p21KO cell line (Fig. 2*D*). The same was observed in a colony formation assay, where the effect of OGT inhibition was much more mild in HT1080 p21KO cells than the parental cell line (Fig. 2*E*). In either cell line, there was no significant change in cell death upon treatment with OSMI-1 (Fig. 2*F*). We repeated the same panel of experiments using a hepatic adenocarcinoma cell line (SK-HEP-1) for which we had CRISPR-Cas9–derived p21KO cells (Fig. S2*C*). Here too, inhibition of OGT decreased cell viability and proliferation of SK-HEP-1 cells, which was partially or completely rescued in SK-HEP-1 cells lacking p21 (Fig. 2, *G* and *H*). Similarly, cell death was not induced by the treatment in either cell line (Fig. 2*I*). Taken together, we conclude that OGT inhibition in certain cancer cell lines leads to reduced cell proliferation without inducing cell death and this is at least partially dependent on p21.

**Figure 3:**
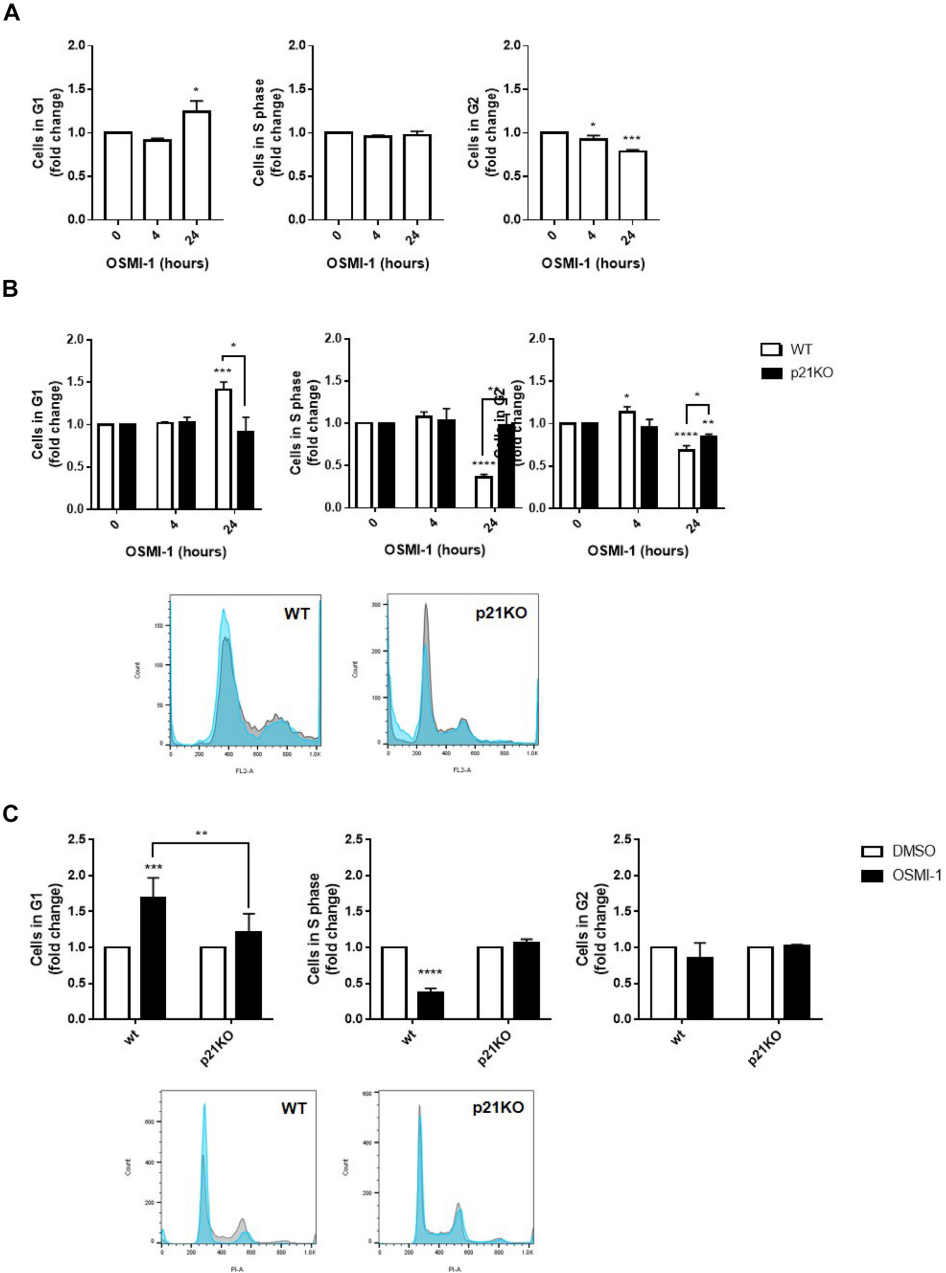
**OGT inhibition induces cell cycle arrest through p21 in a p53-independent manner.***A*, cell cycle analysis of HT1080 p53KO cells in response to treatment with OSMI-1 (50 μM). *B*, cell cycle analysis of HT1080 cells WT (*white bars*) or p21KO cells (*black bars*) in response to treatment with OSMI-1 as in (*A*). *C*, cell cycle analysis of SK-HEP-1 cells WT or p21KO in response to treatment with OSMI-1 for 24 h as in (*A*). Representative histograms show control condition, DMSO, in *gray* and OGT inhibition, OSMI-1, in *blue*. Bar graphs were derived from combined biological replicates. Experiments shown represent at least three biological replicates, ∗*p* < 0.05, ∗∗*p* < 0.01, ∗∗∗*p* < 0.001, ∗∗∗∗*p* < 0.0001. OGT, *O*-GlcNAc transferase.

We hypothesized that the decrease in proliferation upon OGT inhibition shown above could result from induction of cell cycle arrest, which occurred as a result of the increasing levels of p21. Accordingly, we analyzed the cell cycle in response to OGT inhibition and found that, indeed, OGT inhibition led to arrest in both parental and p53KO HT1080 cells, (Fig. 3, *A* and *B*), as well as in parental SK-HEP-1 cells (Fig. 3*C*) and in H1299 cells (Fig. S3*B*). Further, loss of p21 expression completely rescued the effects of OGT inhibition on the cell cycle in both HT1080 p21KO and SK-HEP-1 p21KO cells (Fig. 3, *B* and *C*). These data together show that p21 is a crucial player in mediating the effects of OGT inhibition on cell proliferation, which occurs through the regulation of the cell cycle by p21.

### OGT regulates p21 protein stability independent of p53

*O*-GlcNAcylation modulation in cells harboring WT p53 leads to increased expression of p21 mRNA (22). Since, as mentioned in the introduction, there are p53-independent modes of regulating p21 RNA expression, we measured mRNA levels of p21 in response to inhibition of OGT. As expected, p21 mRNA was induced in HT1080 cells harboring WT p53 in response to OGT silencing; however, in p53KO cells, there was no significant change in p21 mRNA (Fig. S4*A*). Similarly, there was no increase in p21 mRNA in H1299 (p53 *null*) cells when expression of OGT was inhibited by siRNAs (Fig. S4*B*), indicating that p53 is needed for transcriptional activation of the p21 gene in response to inhibition of OGT.

**Figure 4:**
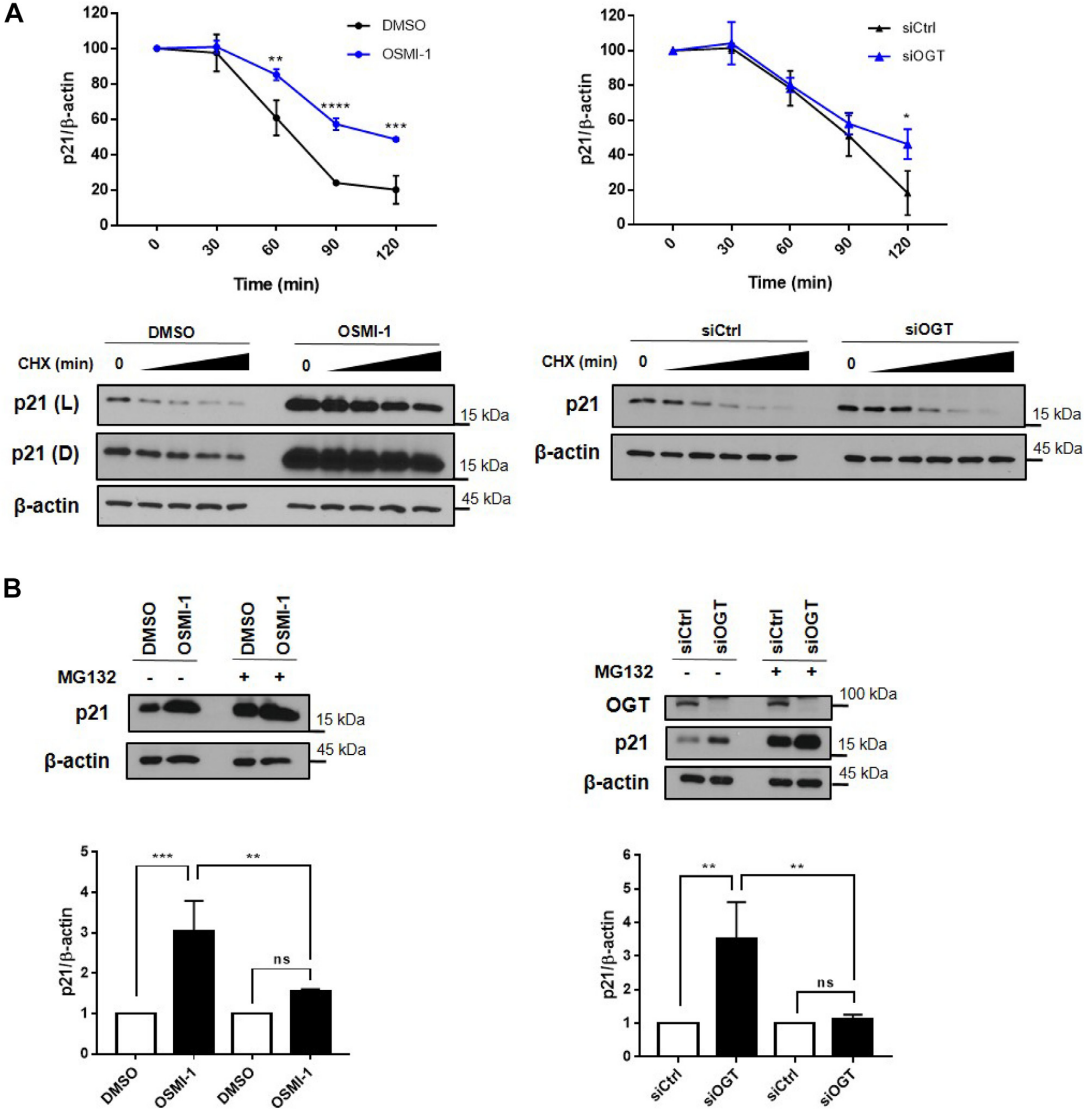
**OGT inhibition regulates p21 protein stability in cells lacking p53.***A*, HT1080 p53KO cells were treated with OSMI-1 (50 μM for 24 h; *left*) or siRNA silencing (for 24 h; *right*) after which cycloheximide (100 μg/ml) was added for the indicated times prior to processing for immunoblotting. Quantification plots and representative blots are shown. Plots correspond to combined biological replicates where protein levels were normalized to β-actin. Representative blots on *left* are shown light (L) and dark (D) exposures of p21 bands. *B*, levels of p21 after OGT inhibition or silencing in response to MG132 (25 μM for 4 h) treatment. Quantification plots correspond to combined biological replicates where protein levels were normalized to β-actin. OGT-inhibiting conditions (OSMI-1 and siOGT) were normalized by their respective controls (DMSO or siCtrl) for comparison. Experiments shown represent at least three biological replicates, ∗*p* < 0.05, ∗∗*p* < 0.01, ∗∗∗*p* < 0.001, ∗∗∗∗*p* < 0.0001. OGT, *O*-GlcNAc transferase.

We then examined p21 protein stability first by treatment of cells with cycloheximide and assaying p21 protein levels over 2 h. The results showed that when HT1080 p53KO cells have OGT inhibited, either by OSMI-1 or by using siRNA, the half-life of the p21 protein was significantly increased (Fig. 4*A*). We surmise that since OSMI-1 was more efficient in boosting p21 levels, the difference in p21 half-life was more dramatic in this condition than in cells where OGT was silenced by siRNA.

These results indicated that OGT inhibition stabilizes p21 protein, possibly by decreasing its degradation by the proteasome. To confirm this hypothesis, we treated cells with the proteasome inhibitor MG132. While, as expected, in the absence of MG132, p21 levels were much higher in OGT-inhibited cells (Fig. 4*B*); in cells where proteasomal degradation was inhibited, there was only a very subtle increase in p21 levels in the OSMI-1–treated cells compared to the vehicle-treated control cells. Similarly, in cells where OGT was silenced by siRNA, the difference between control and OGT siRNA cells that were treated with MG132 was not significant. These results together indicate that inhibition of OGT induces stabilization of p21 protein through impairment of its degradation by the proteasome.

### OGT inhibition stabilizes p21 through the FoxM1–Skp2 pathway

p21 is known to be a target of different ubiquitin complexes, including the SCF-ubiquitin complex that contains Skp2 (11). FoxM1 is a key regulator of Skp2 mRNA transcription (26), and inhibition of OGT was reported to decrease FoxM1 expression and consequentially lower the levels of Skp2 (27). Skp2 is a classic regulator of p27 protein stability and is also involved in p21 stabilization by the formation of the SCF–Skp2 complex (28).

**Figure 5:**
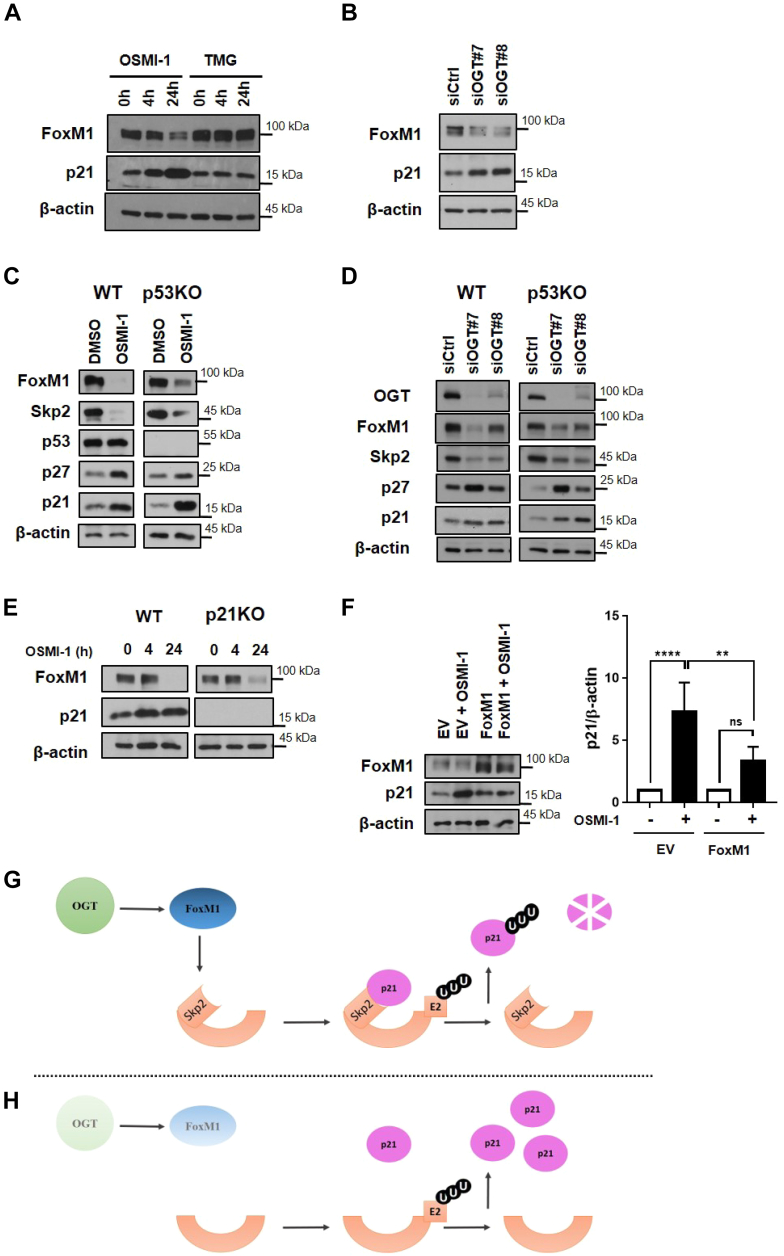
**OGT inhibition increases expression of p21 through downmodulation of the FoxM1-Skp2 axis.***A*, protein levels of FoxM1 and p21 in HT1080 p53KO cells in response to treatment with OSMI-1 (50 μM) or OGA inhibitor, TMG (10 μM) for the indicated times. *B*, protein levels of FoxM1 and p21 in HT1080 p53KO cells in response to OGT silencing using siRNA. *C* and *D*, protein levels of the indicated proteins in HT1080 WT and p53KO cells in response to (*C*) OGT inhibition using OSMI-1 for 24 h or (*D*) OGT silencing using siRNA. *E*, protein levels of FoxM1 in HT1080 parental cells (WT) or p21KO cells in response to treatment with OSMI-1. *F*, protein levels of p21 in response to treatment with OSMI-1 (50 μM) for 24 h in HT1080 p53KO cells overexpressing FoxM1 or empty vector (EV). p21 protein levels were normalized to DMSO controls. Experiments shown represent at least three combined biological replicates ∗*p* < 0.05, ∗∗*p* < 0.01, ∗∗∗*p* < 0.001, ∗∗∗∗*p* < 0.0001. *G* and *H*, model depicting how inhibition or depletion of OGT leads to stabilization of p21 in human cells. *G*, in the presence of OGT, basal levels of FoxM1 lead to expression of Skp2, a component of the SCF-ubiquitin complex. The SCF complex ubiquitinates p21 targeting it to the proteasome for degradation. *H*, in the absence of OGT or decreased OGT activity, the levels of FoxM1 drop dramatically leading to decreased expression of Skp2. The SCF complex lacking Skp2 cannot target p21 for degradation, thereby stabilizing the protein. OGA, *O*-GlcNAcase; OGT, *O*-GlcNAc transferase; SCF, Skp-Cullin-F-box.

We first confirmed that FoxM1 is regulated by OGT in our model, showing that inhibition by either OSMI-1 or siRNA led to decreased levels of FoxM1 (Fig. 5, *A* and *B*). Since FoxM1 upregulates the Skp2 component of the SCF complex, we also determined protein levels of Skp2. Our results showed that when OGT was inhibited by OSMI-1 or siRNAs, FoxM1 as well as Skp2 were decreased while levels of p21 and p27 (both targets of the SCF complex by interaction with Skp2) were increased and this was independent of the presence of p53 (Fig. 5, *C* and *D*).

However, p21 was also shown previously by our group to repress FoxM1 expression under certain conditions (29). We investigated if a decrease in FoxM1 levels is the cause or consequence of increased p21 stability. We determined the levels of FoxM1 protein in both parental HT1080 and HT1080 p21KO cells treated with OSMI-1. In both cell lines, FoxM1 levels were markedly lower by 24 h after treatment with OSMI-1 (Fig. 5*E*). Since this occurred in both cell lines, it is likely that p21 is not regulating FoxM1 levels in this setting.

To extend the findings that FoxM1 is indeed involved in the regulation of p21 by OGT we overexpressed FoxM1 in HT-1080 p53KO cells and examined the impact of OSMI-1 treatment on p21 levels in this situation. Ectopic expression of FoxM1 prevented the increase in p21 levels caused by treatment with the OGT inhibitor (Figs. 5*F* and S5). This result supports our assumption that OGT acts on p21 protein stability by reducing FoxM1 levels and thereby p21 degradation.

## Discussion

We report here that inhibition or silencing of OGT upregulates p21 protein levels by two different mechanisms: one dependent on p53 activation of p21 gene expression and by a second process that is independent of p53, where impairment of p21 protein degradation by the proteasome is the major if not sole means by which p21 is stabilized. We demonstrate as well that the effects of OGT inhibition on p21 levels are responsible for decreasing cell proliferation through the induction of cell cycle arrest.

In an ovarian cancer model, overexpression of both *O*-GlcNAc enzymes, OGA and OGT, leads to activation of p53 and transcription of p53 target genes (22). Here, we found that modulation of OGT but not OGA induces p21 expression in fibrosarcoma cells harboring WT p53. Since OGA modulation did not affect p21 induction by p53 in these cells, our work suggests that the impact of OGA modulation is cell-type specific. In line with this, *O*-GlcNAcylation levels and importance vary among different tissue types and OGT and OGA expression have different impacts and roles in different cancer types (17, 30, 31).

In the current study, we tested more than five different cell types that were derived from blood, muscle, and epithelial sources, and different tumors types (carcinomas and sarcomas) as well as normal-like cells. In all cells tested, OGT inhibition leads to increased p21 levels regardless of the presence of p53, suggesting a common process used by human cells to stabilize p21 protein levels. We observed that treatment with OSMI-1 has a bigger effect in p21 levels than the use of siRNA in different cells. This difference may be due to residual OGT expression after transfection with siRNA against OGT that although reducing the enzyme levels very significantly might not completely oblate its expression. Although the response to OGA inhibition varies among different cell types, our study indicates that blocking OGT is a common pathway in the regulation of p21 protein stability.

The effects of OGT inhibition on cell viability was shown to be primarily due to induction of cell cycle arrest by p21; however, while this arrest was completely rescued by deletion of p21 in HT1080 and SK-HEP-1 cells treated with OSMI-1, cell viability was only partially rescued by the lack of the CDK inhibitor. This indicates that OGT is affecting other pathways that, in conjunction with the FoxM1–p21 axis, lead to decreased cell viability. OGT is known to modulate levels and/or activity of several proteins in cells, many of them involved in cell proliferation and survival (32) and knockdown of OGT leads to decreased proliferation in other models (33, 34). Our work indicates that regulation of p21 is a critical pathway for the regulation of the cell cycle upon OGT inhibition/silencing at least in the three cell lines tested in this work (HT1080, SK-HEP-1, and H1299).

The relationship between OGT inhibition and FoxM1 expression was first reported in a study showing that silencing of OGT reduces FoxM1 levels (27). The authors reported that this regulation of FoxM1 happens at the protein level but FoxM1 is not *O*-GlcNAcylated (27). Subsequently, OGT was shown to regulate FoxM1 ubiquitination through SIRT1 (35). Building on these findings, we found that rescue of FoxM1 levels in OGT-inhibited cells prevents the stabilization of p21. Although Caldwell *et al.* correlated decreased FoxM1 expression with accumulation of p27, they did not show the effects in p21. We show in our model that both proteins accumulate in response to OGT-mediated downregulation of FoxM1. Further, although both members of the Cip/Kip family regulate the cell cycle similarly, in our model, using HT1080 and SK-HEP-1 cells, p21 is the main functional factor involved in the regulation of the cell cycle.

Whether OGT inhibition has cancer relevance in physiological settings is still somewhat unclear. Silencing of OGT was shown to decrease tumor burden (27, 35), which was partially rescued by inhibiting FoxM1 depletion by SIRT1 (35), showing the importance of the pathway to disease progression.

Clinically, our data are relevant to studies on aging, cancer, chronic obstructive pulmonary diseases, diabetes, and neural degeneration among other pathologies. For instance, senescence was shown to be an important driving mechanism in chronic obstructive pulmonary disease (36) and this process is regulated by p21. Of relevance to cancer treatment, we propose that OSMI-1 be considered as a possible candidate for adjuvant therapy due to its apparent lack of cytotoxicity in mice (24) as well its reported effects on tumor volume reduction (37, 38). In line with this, since p21 was recently reported to induce immune surveillance (39), OSMI-1 may have additional benefits for cancer treatment. Although investigation of possible off-target effects of the drug and more specific cytotoxicity analysis are needed, future studies to examine these possibilities are strongly encouraged.

In conclusion, we have expanded a previously described pathway for induction of p21 expression by modulation of *O*-GlcNAcylation through p53 activation. Previously, OGT was shown to regulate levels of p21 mRNA (22), and we now add another level of regulation of p21 expression that works through protein stabilization (Fig. 5, *G* and *H*). Additionally, we discovered a new pathway by which OGT regulates p21 protein levels independent of p53, through the FoxM1–Spk2 axis; this pathway was seen in both naturally occurring p53 null cells and CRISPR-engineered cells that cannot express p53 protein and appears to be the main form of regulating the cell cycle by OGT in different cells.

## Experimental procedures

### Chemicals and reagents

All primary and secondary antibodies for immunoblotting were used at 1:1000 and 1:2000 dilutions, respectively. The following reagents were used for the experiments in this study: anti-*O*-linked-N-acetylglucosamine antibody (RL2) (MA1-072) and anti-OGT (PA5-22071) were from Thermo Fisher Scientific; anti-b-actin (A2066), anti-rabbit-HRP (A6154), and anti-mouse-HRP (A4416) were from Sigma-Aldrich; anti-p21 (2947S), anti-p27 (3686S), and anti-Skp2 (4358S) were from Cell Signaling; and anti-FoxM1 (sc-502) was from Santa Cruz. TMG (SML-0244), OSMI-1 (SML-162), and cycloheximide (C4859) were purchased from Sigma-Aldrich. siRNAs against OGT #7 (SI02665131) and #8 (SI04713604) were purchased from Qiagen, and siRNA #1 (SI6094) and siCtrl siRNA (4390843) were purchased from Ambion. A FoxM1 overexpression plasmid (sc-128214) and empty vector control plasmid were purchased from OriGene. Transfection of plasmids was performed using Mirus TransIT X2 transfection reagent, and siRNAs were transfected using Lipofectamine RNAiMax (Thermo Fisher).

### Cell culture

Human cancer cell lines HT1080, A549, MCF-7, SK-HEP-1, PANC-1, and MIA PaCa-2 were grown in Dulbecco’s Modified Eagle’s Medium (12100-061, Thermo Fisher Scientific) supplemented with 10% heat-inactivated fetal bovine serum, 100 units of penicillin, and 100 mg/ml streptomycin. HT1080 and SK-HEP-1 cells were also supplemented with nonessential amino acids (11140050, Gibco). MIA PaCa-2 cells were also supplemented with 2.5% heat-inactivated horse serum (H1138, Millipore Sigma). THP-1, BxPC3, and H1299 cells were grown in RPMI-1640 supplemented with 10% fetal bovine serum, 100 units of penicillin, and 100 mg/ml streptomycin. THP-1 was also supplemented with 0.05 mM β-mercaptoethanol. All cell lines were maintained at 37 °C with 5% CO2. p53 and p21 KO HT1080 and SK-HEP-1 stable cell lines were generated using CRISPR-Cas9 technology as described previously (40, 41).

### Immunoblotting

Cells were washed with phosphate-buffered saline and homogenized in lysis buffer (120 mM NaCl, 50 mM, Tris–HCl, pH 7.4, 1 mM EDTA, 1% NP-40, 0.25% deoxycholate Na, 1 mM PMSF) with the addition of protease inhibitors, phosphatase inhibitor, and OGA inhibitor (TMG, 1 μM). Protein concentrations were determined by the Bradford assay. Laemmli buffer was added to samples which were boiled for 2 min. Samples were separated on SDS-polyacrylamide gels and subsequently transferred to PVDF membrane (Invitrogen). The membranes were blocked in Tris-buffered saline with 0.1% (v/v) Tween 20 and 3% (w/v) bovine serum albumin. The blocked membranes were then incubated overnight at 4 °C with primary antibodies. Blots were washed and then incubated with the appropriate secondary antibody for 1 h, prior to visualization using chemiluminescent horseradish peroxidase reagent (32106, Thermo Fisher Scientific) according to manufacturer’s instructions. ImageJ software (https://imagej.nih.gov/ij/) was used for densitometry analysis of immunoblots, and measurements were normalized against b-actin as a loading control.

### Quantitative real time PCR

Total RNA was isolated using Qiagen RNeasy kit according to the manufacturer’s instructions. A total of 1 μg of input RNA was used to generate cDNA using the Qiagen Quantitect reverse transcription kit. The cDNA products were diluted (1:10) with nuclease-free water and analyzed by qPCR using SYBR Green dye (Thermo Fisher Scientific) according to the manufacturer’s instructions. Quantitative PCR analysis was performed using a AB StepOnePlus real time PCR detection system (Thermo Fisher Scientific) with the following protocol: polymerase activation and DNA denaturation for 30 s at 95 °C; amplification denaturation for 5 s at 95 °C and annealing for 30 s at 62 °C with 40 cycles; and melt curves were done at 65 to 95 °C with 0.5 °C increments (5 s/step). Quantification cycle values were recorded by StepOne software. Relative changes in cDNA levels were calculated using the comparative Ct method (ΔΔCT method). Primers used for *CDKN1A* detection are as follows: forward, 5′-GCAGACCAGCATGACAGATTT-3′ and reverse, 5′-GGATTAGGGCTTCCTCTTGGA-3′. *RPL32* was used as a housekeeping gene control using the following primers: forward, 5′-TTCCTGGTCCACAACGTCAAG-3′ and reverse, 5′-TGTGAGCGATCTCGGCAC-3′.

### Cell cycle and DNA fragmentation analysis

Cell cycle was analyzed by distribution of cells stained with propidium iodide (PI) (P4170, Sigma-Aldrich). After the desired treatment/transfection, cells were harvested and fixed with 1:1 PBS-ethanol for 24 h. Then, samples were washed with PBS and resuspended in a PI solution (66 μg/ml PI, 100 μg/ml RNAse A) for 15 min in the dark at 37 °C. Cells were analyzed by flow cytometry (FL-2A) (BD Celesta, BD Biosciences). Data acquisition and analysis were obtained using BD FACSDiva software (BD Biosciences).

### Clonogenic assay

HT1080 and SK-HEP-1 parental, p21KO and p53KO cell were seeded at 3000 cells/well in 6-well plates and maintained overnight prior to treating with DMSO or OSMI-1 (50 μM) for 24 h. After treatment, culture dish wells were washed with PBS and replaced with new media. Cells were maintained in culture for 7 days. H1299 cells were transfected with ctrl siRNA or different OGT siRNAs for 24 h and then cells were trypsinized and reseeded at 5000 cells/well in 6-well plates. Cells were maintained in culture for 9 days. Colonies formed after the determined periods of time were stained with Crystal Violet (C3886, Sigma-Aldrich).

### Viability assay

Cells were plated in 96-well plates (1.5 × 10^4^ cells/well) and treated with DMSO or OSMI-1 (50 μM) for 4 or 24 h. The viability of cells was measured by adding a 1:1 dilution of CellTiter-Glo luminescent reagent (G7573, Promega) with media followed by shaking for 2 min and then 10 min incubation at room temperature. The intensity of luminescence was determined using a plate reader which was normalized to DMSO controls.

Quantification of dead cells was performed by using SYTOX Green (S34860, ThermoFisher). Briefly, cells under different experimental conditions were collected in Tris-buffered saline and incubated with SYTOX Green for 10 min at room temperature. The dye was washed out and cells were analyzed by flow cytometry (BD Celesta, BD Biosciences). Data acquisition and analysis were obtained using by BD FACSDiva software (BD Biosciences).

### Statistical analysis

All data reported in this paper were expressed as the mean ±SD derived from at least three independent experiments. A significant difference from the respective control for each experimental test condition was assessed by two-way or one-way analysis of variance or Student’s *t* test using GraphPad Prism 7.0 software. Values of *p* < 0.05 were considered statistically significant. In all figures, ∗*p* < 0.05, ∗∗*p* < 0.01, ∗∗∗*p* < 0.001, ∗∗∗∗*p* < 0.0001.

## Data availability

This study includes no data deposited in external repositories.

## Supporting information

This article contains supporting information.

## Conflict of interest

The authors declare that they have no conflicts of interest with the contents of this article.
