# Supplementary material for: O-GlcNAc transferase regulates p21 protein levels and cell proliferation through the FoxM1–Skp2 axis in a p53-independent manner
Source: J Biol Chem. 2022 Jul 20;298(9):102289. doi: 10.1016/j.jbc.2022.102289 (PMC9418910; doi:10.1016/j.jbc.2022.102289)
Supplement: Supplemental Figures S1–S5 [file mmc1.pdf]

**Supplementary Information**

# *O*-GlcNAc tranferase regulates p21 protein levels and cell proliferation through the FoxM1-Skp2 axis in a p53-independent manner

Rafaela Muniz de Queiroz, Sung-Hwan Moon and Carol Prives

List of content:

Figure S1……………………………………………………………………….page S2
Figure S2……………………………………………………………………….page S3
Figure S3……………………………………………………………………….page S4
Figure S4……………………………………………………………………….page S4
Figure S5……………………………………………………………………….page S5

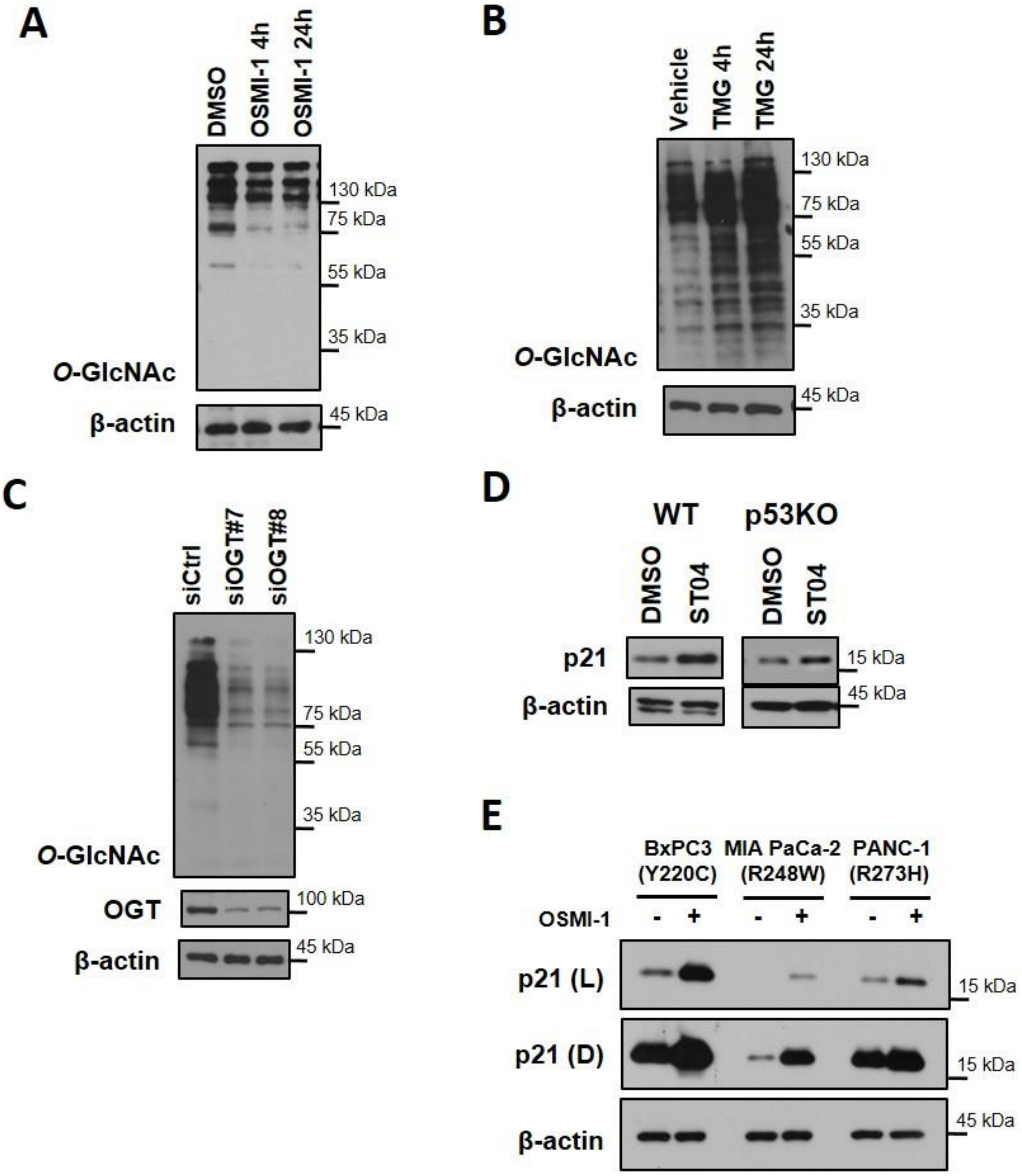


**Figure S1: OGT inhibition induces decreased O-GlcNAcylation and increased p21 levels.** Global O-GlcNAcylation levels in response to (A) 50μM of OSMI-1 or (B) 10μM of TMG for 24 h and (C) silencing of OGT using siRNAs in HT1080 p53KO cells. Note that in B extracts that were processed for immunoblotting are from the same gel as that shown in Fig. 1A, HT1080 p53KO cells treated with TMG. Note that in C extracts of that were processed for immunoblotting are from the same gel as that shown in Fig 1B. (D) p21 levels in response to OGT inhibitor ST045849 (ST04) after 24 h treatment in HT1080 WT and p53KO cells. (E) p21 levels in response to OSMI-1 after 24 h treatment in cells harboring mutant p53. Representative blots are shown light (L) and dark (D) exposures of p21 bands. The experiments shown represent at least 3 biological replicates.

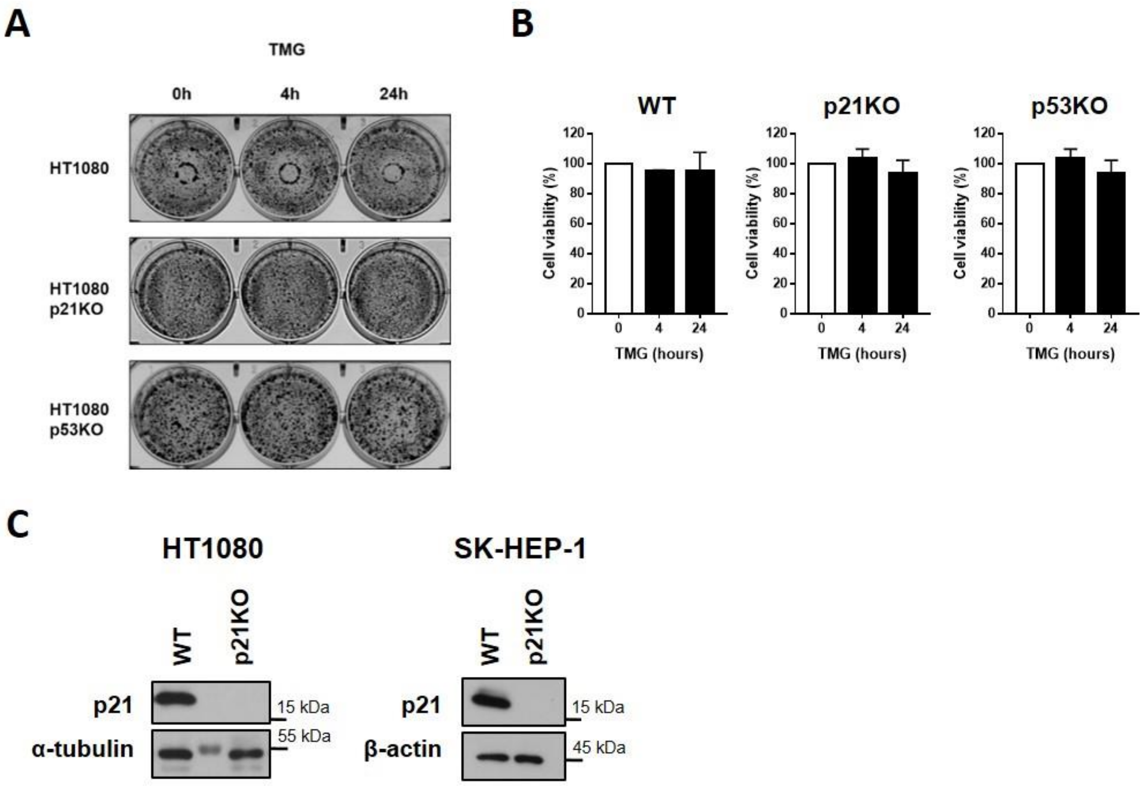


**Figure S2: Cell viability and proliferation is not affected by OGA inhibition.** (A) Cell proliferation in response to OGA inhibitor, TMG, in HT1080 cells (WT, p21KO and p53KO). (B) Viability of HT1080 cells (WT, p21KO and p53KO) in response to TMG. Quantification plots correspond to different biological replicates combined. (C) Confirmation of p21 knockdown in HT1080 and SK-HEP-1 cell line. p21 levels in SK-HEP-1 or HT1080 WT and p21KO cells. The experiments shown represent at least 3 biological replicates

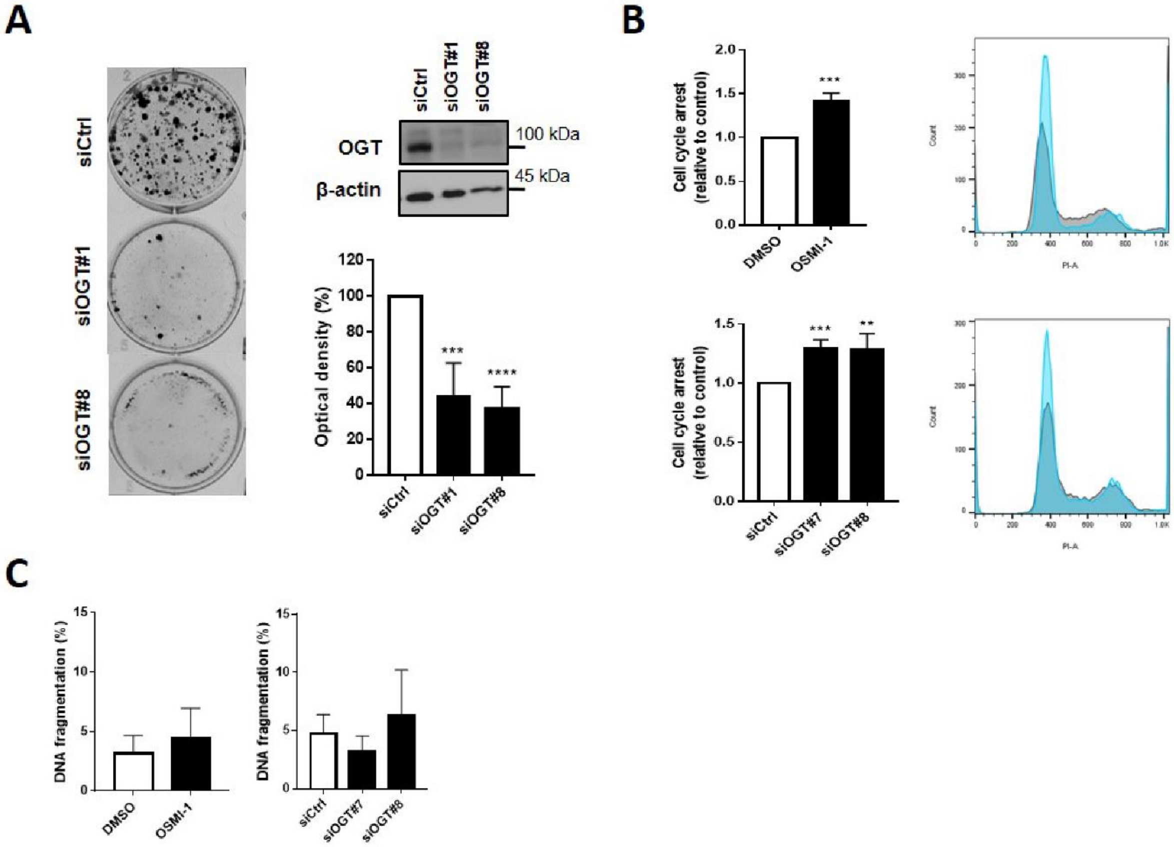


**Figure S3: OGT inhibition reduces proliferation and induces cell cycle arrest but does not affect cell death in H1299 cells.** (A) Cell proliferation in response to silencing of OGT using siRNA. (B) Cell cycle analysis in response to OGT inhibition using OSMI-1 and siRNA. Representative histograms show control condition, DMSO or siCtrl, in grey and OGT inhibition, OSMI-1 or siOGT#7, in blue. (C) Quantification of cell death in response to OGT inhibition using OSMI-1 and siRNA. Graph represents percentage of cell population exhibiting DNA fragmentation detected by PI staining. The experiments shown represent at least 3 biological replicates. **p<0.01, ***p<0.001. ****p<0.0001.

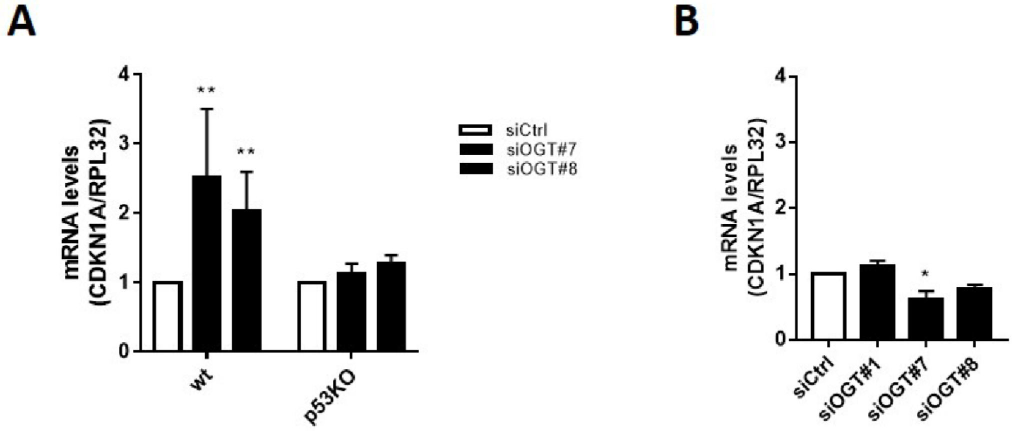


**Figure S4: OGT induces p21 gene expression in a p53 dependent manner.** (A) Quantification of mRNA levels of p21 in response to OGT silencing in HT1080 cells harboring

wild-type p53 (WT) or in p53KO cells. (B) Quantification of mRNA levels of p21 in response to OGT silencing in H1299 cells (p53 *null*). The experiments shown represent at least 3 biological replicates. *p<0.05, **p<0.01.

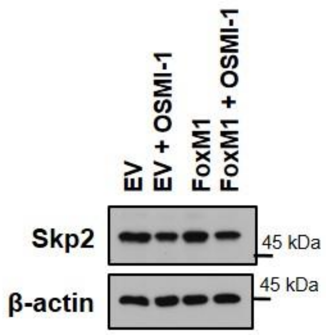


**Figure S5: Skp2 levels in response to treatment with OSMI-1 in cells overexpressing FoxM1.** Protein levels of Skp2 in cells treated with OSMI-1 for 24h overexpressing either empty vector (EV) or FoxM1. β-actin was used as loading control. The experiment shown represents 3 biological replicates.
